# Does mammalian target of rapamycin or sestrin 1 protein signaling have a role in bone fracture healing?

**DOI:** 10.3906/sag-1809-117

**Published:** 2019-12-16

**Authors:** Özhan PAZARCI, Halef Okan DOĞAN, Seyran KILINÇ, Yalkin ÇAMURCU

**Affiliations:** 1 Department of Orthopedics and Traumatology, Faculty of Medicine, Cumhuriyet University, Sivas Turkey; 2 Department of Biochemistry, Faculty of Science, Cumhuriyet University, Sivas Turkey; 3 Department of Orthopedics and Traumatology, Faculty of Medicine, Erzincan University, Erzincan Turkey

**Keywords:** mTOR, sestrin, bone, fracture

## Abstract

**Background/aim:**

Fracture healing is a complex physiological process that involves a well-orchestrated series of biological events. The mammalian target of rapamycin (mTOR) and sestrin 1 (SESN 1) play a central role in cell metabolism, proliferation, and survival. The aim of our study is to present serum mTOR and SESN 1 levels by comparing patients with or without bone fractures. It is also a guide for further research on the roles of these proteins in fracture healing.

**Materials and methods:**

A total of 34 patients (10 females, 24 males) with bone fractures and 32 controls (10 females, 22 males) participated in this study. After collecting serum venous blood samples, the quantitative sandwich ELISA technique was used for the determination of serum mTOR and SESN 1 levels.

**Results:**

The mean serum mTOR level was significantly higher in the fracture group compared to the control group (P = 0.001). However, SESN 1 levels did not significantly differ between groups (P = 0.913).

**Conclusion:**

We found that serum mTOR levels increased on the first day after fracture compared to the control group. However, we obtained no significant difference between groups in terms of SESN 1 levels. This study may guide further clinical studies investigating the potential role of mTOR signaling in the bone healing process.

## 1. Introduction 

Fractures, mostly arising from injury, are an important public health burden [1]. Approximately 5%–10% of the 6.2 million fractures occurring annually in the United States are associated with impaired healing including delayed healing or nonunion [2]. 

Fracture healing is a complex physiological process that involves a well-orchestrated series of biological events [3]. While our knowledge has vastly expanded, with the increasing understanding of the multiple factors and complex pathways involved, many new developments are anticipated in the years to come. Many local and systemic regulatory factors such as growth and differentiation factors, hormones, cytokines, and the extracellular matrix interact with several cell types including bone and cartilage, forming primary cells or even muscle mesenchymal cells, recruited at the fracture-injury site or from circulation [4].

The mammalian target of rapamycin (mTOR) protein, a 289-kDa serine-threonine kinase, has a central role in cell metabolism, proliferation, and survival [4,5]. Different factors including nutrients, growth factors, cellular energy, and stress regulate mTOR signaling [6]. Insulin and Ras (Rat sarcoma) signaling pathways are also important regulators of mTOR signaling activation [7]. The role of the mTOR pathway has been examined in age-related pathologies such as neurodegenerative disease, cancer, heart disease, and metabolic diseases [8–11]. Recent studies have implicated the potential role of mTOR in regulating multiple aspects of cartilage development and preosteoblast differentiation [12,13]. 

Sestrins (SESN) are highly conserved proteins that exhibit oxidoreductase activity and therefore the expression of SESNs is upregulated by DNA damage, oxidative stress, and hypoxia [14]. Independent of their antioxidant activity, SESNs are also negative regulators of mTOR signaling. Several studies revealed the importance of SESNs in insulin resistance, muscle degeneration, cardiac dysfunction, mitochondrial pathologies, and tumors, but little is known about the relationship between bone fractures and mTOR and SESN-1. 

Previous studies have emphasized the role of mTOR signaling in bone metabolism [2]. However, to date, we could find no clinical study investigating serum mTOR and SESN-1 concentrations in patients with the bone fracture. We hypothesized that defining serum mTOR and SESN-1 concentrations in patients with fractures may demonstrate their potential roles in the bone healing process. The evaluation of the relationship between these serum proteins and the pathway occurring in response to bone fracture may also guide further studies describing the bone healing process. Therefore, in this study, we aimed to compare serum mTOR and SESN-1 levels in patients with or without bone fracture.

## 2. Materials and methods

### 2.1. Study population

This cross-sectional study was completed after approval from our institutional ethical review board and was conducted in accordance with the Declaration of Helsinki. Informed consent was obtained from each participant.

Between May 2017 and October 2017, patients who were admitted to our university hospital and diagnosed with bone fracture were included in the study. Patients who were over 65 years old (52 patients) or had systemic comorbid diseases (25 patients) were excluded from the study. Patients with high-energy trauma (30 patients) and multiple fractures were also excluded from the study group. A control group consisting of healthy individuals between 18 and 65 years of age was created by a biochemistry laboratory physician who selected participants from consecutive healthy individuals. A total of 34 patients (10 females, 24 males) with bone fractures and 32 controls (10 females, 22 males) participated in the study.

### 2.2. Blood sample collection and analysis 

In the fracture group, overnight fasting venous blood samples were taken from the patients on the first day of stay in the hospital. The control group consisted of healthy individuals between 18 and 65 years old without any comorbid disease and bone fracture. These individuals were called 1 day before and asked to attend after overnight fasting. Venous blood samples were collected from all participants into red-top tubes (Becton Dickinson, UK). The serum sample tubes were allowed to clot before centrifugation. After centrifugation at 4 °C for 15 min at 3500 rpm, the serum was aliquoted and immediately frozen at ˗80 °C (WiseCryo, South Korea). The quantitative sandwich ELISA technique was used for the determination of serum mTOR and SESN-1 (YL Biotech Co. Ltd., China). Tests were performed according to the manufacturer’s recommendations. Patients with insufficient samples or incorrect results were excluded from the study.

### 2.3. Statistical analysis

The sample size estimation was calculated using GPower software. The minimum sample size was calculated by taking into account the large effect size of results for a false-positive rate of 5% (*α *= 0.05) and a power of at least 80% (*β *= 0.2) [15–17]. Using these parameters and adjusting for multiple comparisons, we required a minimum sample size of 26 patients for each group. A total of 32 patients per group was requested, taking into consideration the estimated 20% dropout rate (failure of test results); thus, a total of 64 patients were planned to be enrolled in the current study.

Statistical analysis was performed using SPSS 22.0 (IBM Corp., USA). Numerical variables are given as means and standard deviations, and categorical variables are given as frequencies and percentages. The comparison of means was completed with the t-test or Mann-Whitney U-test in accordance with the Shapiro–Wilk normality test. Comparison of frequencies was performed with the chi-square test and the Pearson correlation test was used to evaluate the correlation coefficient between mTOR and SESN-1 levels in both study groups. P < 0.05 was considered to be statistically significant. 

## 3. Results

A total of 66 patients (46 females, 20 males) were enrolled in the study. The fracture group consisted of 34 patients (24 females, 10 males) with mean age of 45.8 ± 12.6 years (range: 18 to 64 years). The control group consisted of 32 individuals (22 females, 10 males) with mean age of 47.6 ± 10 years (range: 28 to 63 years). The fracture group and control group had no significant differences in terms of age or sex (P = 0.728 and P = 0.334, respectively). The mean serum mTOR level was significantly higher in the fracture group compared to the control group (P = 0.001). However, SESN-1 levels did not significantly differ between groups (P = 0.913) (Table 1). The distributions of fractures in the fracture group are shown in Table 2.

**Table 1:** mTOR and SESN-1 levels of the study groups.

ng/mL	Fracture group (n = 34)	Control group (n = 32)	P-values
mTOR	15.4 ± 6.2 (0.1–22.9)	9.6 ± 5.8 (0.1–22.9)	0.001*
	[13.2–17.5]	[7.2–12.1]	
SESN-1	19.4 ± 8.7 (0.2–33.8)	19.6 ± 7.3 (0.2–28)	0.913*
	[16.4–22.4]	[16.5–22.7]	

* P-values according to Student t-test.

**Table 2:** The distribution of fractures.

Bone	n	%
Humerus	3	8.8
Hand/forearm	2	5.9
Pelvis	4	11.8
Hip	2	5.9
Femur	5	14.7
Tibia	5	14.7
Foot/ankle	5	14.7
Others	8	23.5
Total	34	100

We observed a significant correlation between mTOR and SESN-1 levels in the fracture group (P = 0.015, r = 0.412) and in the control group (P = 0.043, r = 0.359). 

## 4. Discussion

Bone fracture is a common injury that may initiate a series of physiological and pathological reactions. A number of promising therapeutic approaches have been developed, such as improvement of internal fixation devices and the application of novel biological materials; however, delayed healing or nonunion may occur in 5%–10% of fractures, adding further to patient morbidity and the expense of treatment [18]. The improvement of patient morbidity and reduction of costs is an incentive for the development of novel therapies to enhance fracture healing [19]. Recent studies have indicated the complexity of the biological structure of bone [20]. The most important finding of this study was that serum mTOR levels increased with fractures while serum SESN-1 levels remained stable when patients with fractures were compared with a control group. 

mTOR is identified as a key cellular signaling protein used to respond to diverse environmental stimuli, control numerous processes that generate or consume a mass of energy and nutrients, regulate most major cellular functions, and assist most organisms’ inefficient transformation between anabolic and catabolic states. It plays an important role in regulating basic cell behaviors like growth and proliferation and also has an important role in bone growth and proliferation [21]. On the other hand, there is still debate about the effect of mTOR in osteogenesis. The negative and positive effects of mTOR inhibition on osteogenesis were also reported [21,22]. Interestingly, the activation of mTOR signaling has also been found to promote osteoblast differentiation [21]. In summary, studies showed that mTOR signaling serves as a double-edged sword in regulating cell differentiation [21]. These differences appear to depend on cell type and treatment conditions used [22]. Holstein et al. reported that rapamycin, an mTOR inhibitor, affected early fracture healing and inhibited callus formation in rats [16]. In another study by Yang et al., the authors mentioned that the inhibition of mTOR promoted bone fracture healing in rats [17]. In our study, mTOR levels were significantly higher in the fracture group compared to the control group. 

Departing from in vitro and animal studies, we analyzed serum mTOR levels in our patients and found significantly higher mTOR levels. However, it should be kept in mind that stress and inflammatory response secondary to trauma may also lead to the increased mTOR levels. After fracture, the bone healing process begins immediately with increases in protein and growth factor synthesis, which play roles in fracture healing [23]. Previous studies showed the contribution of mTOR signaling to protein synthesis [24]. The increased levels of mTOR in our patient group may be due to increased protein synthesis secondary to fracture and trauma. In summary, the current literature agrees on the vital role of mTOR signaling for bone hemostasis [21]. 

In terms of demonstration of mTOR levels in fracture patients, this pathway has been investigated in terms of musculoskeletal disorders after abruptly leaving the mTOR pathway. The effect and significance of mTOR had been previously studied in various areas in the literature such as neurofibromatosis, rotator cuff tears, pathologies of nucleus pulposus, and osteosarcoma [25–29]. We think that this will be a guide for future studies about fractures in our work. 

The SESNs are a class of proteins that are also induced by stress [14]. Currently, three isoforms of the SESN family are known: SESN-1, SESN-2, and SESN-3. Studies have shown that these proteins are important for the maintenance of metabolic homeostasis, for the protection of cells against age-related physiological damage, and, mainly, for the control of adenosine monophosphate kinase (AMPK)/mTOR signaling [30]. Previous studies showed that inhibition of SESN resulted in increases of age- and obesity-related pathologies [14]. SESNs suppress oxidative stress and regulate autophagy [31]. Some conditions have been shown to increase SESN levels such as hypoxia, oxidative stress, DNA damage, and physical exercise [32]. In our study, we also evaluated levels of serum SESN-1 because of its relationship with mTOR signaling. According to our results, we found no significant difference in SESN-1 levels when patients with fractures were compared to healthy individuals. However, some studies reported that SESN overexpression potently suppresses mTOR signaling [30]. We found that there was a correlation between mTOR and SESN in both groups. This shows that more studies are needed to evaluate serum SESN levels on different days after fracture and to examine more patients.

The main limitation of this study is that serum mTOR and SESN-1 levels were only measured in the first 24 h after fracture. However, this study is the first in the literature evaluating serum mTOR and SESN-1 levels in patients with or without fracture. We also compared two homogeneous patient populations without any systemic comorbid disease to exclude other pathologies that may affect mTOR and SESN signaling pathways. 

According to our results, the serum mTOR levels increased the first day after fracture compared to a control group. However, we obtained no significant difference between groups in terms of SESN-1 levels. This study may guide further clinical studies investigating the potential role of mTOR signaling in the bone healing process.

## Acknowledgment

This work was supported by the Scientific Research Projects Fund of Cumhuriyet University under Project Number T724.
